# Protective effect of *Luffa cylindrica* fermentation liquid on cyclophosphamide-induced premature ovarian failure in female mice by attenuating oxidative stress, inflammation and apoptosis

**DOI:** 10.1186/s13048-024-01353-z

**Published:** 2024-01-25

**Authors:** Yueying Feng, Wei Zhang, Xiaowei Xu, Wanzhen Wang, Yuanyuan Xu, Mengqi Wang, Jinfeng Zhang, Hengyi Xu, Fen Fu

**Affiliations:** 1 The Second Affiliated Hospital of Nanchang University, Nanchang University, No. 1 Mingde Road, Nanchang, 330000 People’s Republic of China; 2 State Key Laboratory of Food Science and Resources, Nanchang University, 235 Nanjing East Road, Nanchang, 330047 People’s Republic of China

**Keywords:** *Luffa cylindrica* fermentation liquid, Premature ovarian failure, Cyclophosphamide, Oxidative stress, Inflammation, Apoptosis

## Abstract

Premature ovarian failure (POF) is a leading cause of women’s infertility without effective treatment. The purpose of this study was to investigate the protective effects of *Luffa cylindrica* fermentation liquid (LF) on cyclophosphamide (CTX) -induced POF in mice and to preliminarily investigate the underlying mechanisms. Thirty-two Balb/c mice were divided into four groups randomly. One group served as the control, while the other three received CTX injections to establish POF models. A 14-day gavage of either 5 or 10 μL/g LF was administered to two LF pretreatment groups. To analyze the effects of LF, the ovarian index, follicle number, the levels of serum sex hormones, superoxide dismutase (SOD), glutathione (GSH), malondialdehyde (MDA), inflammatory factors, and apoptosis of the ovarian cells were measured. The effects of LF pretreatment on the expression of TLR4/NF-κB and apoptosis pathways were also evaluated. We found that LF pretreatment increased the ovarian index and the number of primordial and antral follicles while decreasing those of atretic follicles. LF pretreatment also increased the serum levels of estradiol (E2) and anti-Müllerian hormone (AMH), while decreasing those of luteinizing hormone (LH) and follicle-stimulating hormone (FSH). Furthermore, LF pretreatment increased the levels of SOD and GSH in the ovaries, while decreasing those of MDA, tumor necrosis factor-α (TNF-α) and interleukin-1β (IL-1β). LF administration reduced the amount of TUNEL+ ovarian cells and the levels of TLR4 and NF-κB P65 protein expression. In conclusion, LF has antioxidant, anti-inflammatory as well as anti-apoptotic effects against CTX-induced POF, and the inhibition of TLR4/NF-κB and apoptosis pathways may be involved in its mechanisms.

**Graphical Abstract:**

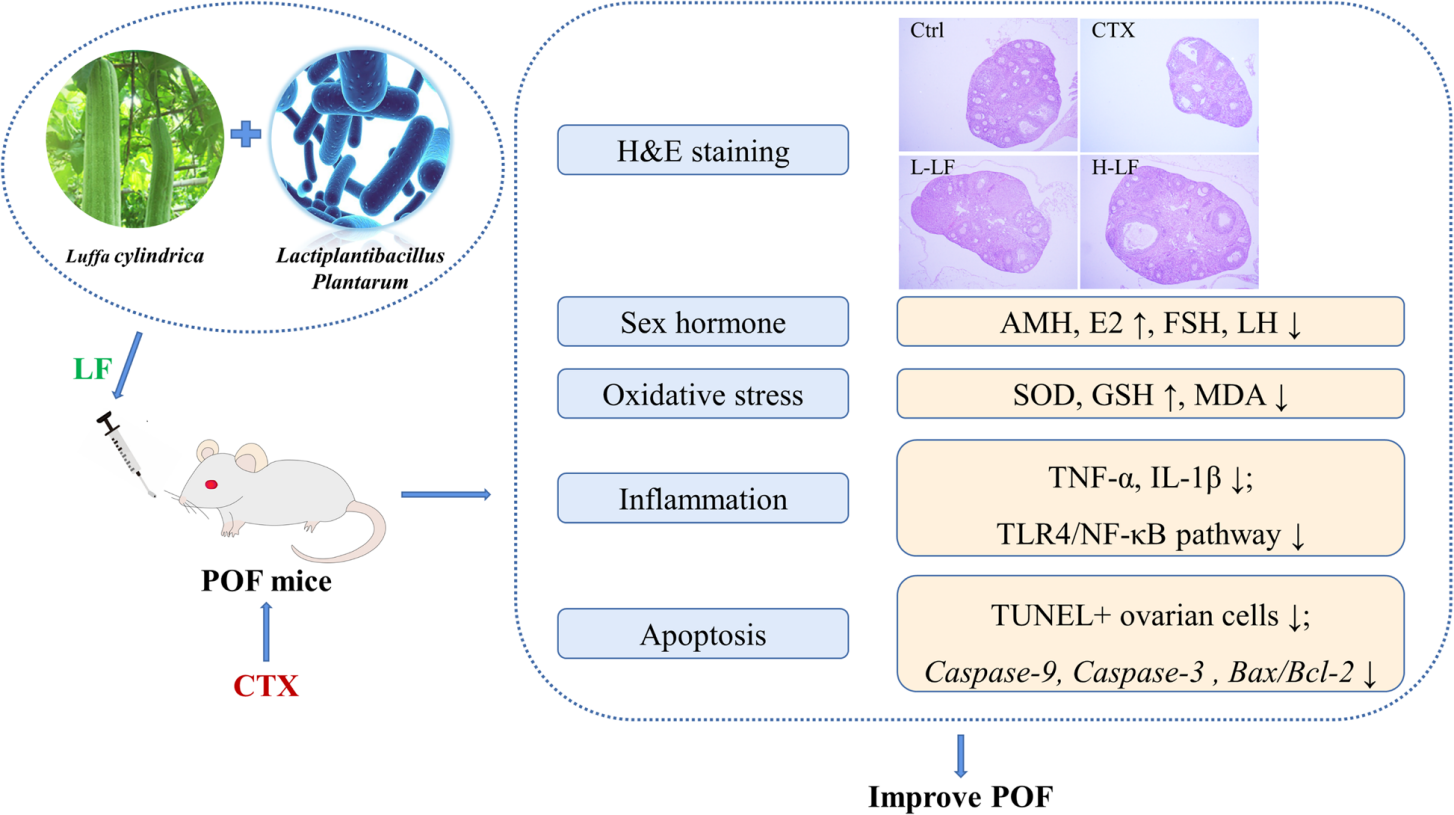

## Introduction

Premature ovarian failure (POF) is the cessation of ovarian function in women before the age of 40. The incidence of POF in recent years is approximately 1% of women according to statistics [1]. POF is mainly manifested by the high levels of gonadotropins and follicle stimulating hormone (FSH), and low level of estradiol (E2), which can lead to amenorrhea and even infertility in severe cases [2, 3], and seriously affect the quality of life and physical and mental health of women. The etiology of POF is complex and the mechanisms are still unclear. Current studies have shown that the common reasons are autoimmunity, genetics, environmental factors, and medical factors (such as surgery, radiotherapy, and chemotherapy) [4].

Chemotherapy is an important method in the treatment of various tumors and immune diseases. However, chemotherapeutic drugs are less selective in targeting tissues and cells. Therefore, while chemotherapy kills the diseased cells, there will be varying degrees of damage to normal tissue and cells, which has become an important reason for POF [5]. Currently, there are increasing reports of ovarian dysfunction and infertility in women due to chemotherapy, which has become a hot topic of discussion in today’s generation, particularly among young infertile female patients [6]. In this circumstance, it is urgent to seek effective methods to prevent or alleviate chemotherapy-induced POF. The classical chemotherapeutic agent cyclophosphamide (CTX) is a commonly used alkylating agent for chemotherapy in clinical oncology patients. CTX can induce non-cyclic toxic effects in cells that have severe reproductive toxicity [7, 8]. Therefore, CTX has been commonly used in previous studies to induce POF in animal models [9–11].

In recent years, lactic acid bacteria fermentation products are gradually being popular because of their rich nutritional value and multiple probiotic effects including antioxidant, antiviral, anti-inflammatory, and immunomodulatory [12]. Some lactic acid bacteria fermented fruits and vegetables have been shown to reduce immune dysfunction, intestinal microbiota disorder and mucosal barrier damage in mice caused by CTX [13, 14]. *Luffa cylindrica*, a member of the Cucurbitaceae family, is a popular daily vegetable that is grown all over the world. *Luffa cylindrica* is rich in vitamins, polyphenols, flavonoids, saponin, triterpenoids, and other nutrients, according to bioactive component analysis [15, 16]. In addition, it has significant biological activities in anti-inflammatory, antioxidant, immunomodulation, and anti-cancer [15, 17–19]. Therefore, we hypothesized that lactic acid bacteria fermented *Luffa cylindrica* might be effective in promoting human reproductive health. However, it is still unknown whether *Luffa cylindrica* fermentation liquid (LF) could be effective in the prevention and treatment of CTX-induced ovarian damage.

Therefore, the purpose of this research was to observe the putative protective effects of LF on ovarian damage in the POF mouse model caused by CTX and to investigate its possible mechanism, which is significant for developing the edible value of LF to protect ovarian function.

## Materials and methods

### Preparation of *Luffa cylindrica* fermentation liquid

Over-mature *Luffa cylindrica* was provided by Jiangxi Meier loofah Co., Ltd. (Jiangxi, China). *Lactiplantibacillus Plantarum* P101 was obtained from previous isolation by our laboratory, and the strain has been kept in the China Type Culture Conservation Center since 2021 under the conservation number CCTCC M 2021108. The *Luffa cylindrica* was washed and cut into pieces and added to distilled water at 1:2 (W/V), then sealed and sterilized at 105 °C for 20 min to obtain a sterile loofah mixture. After the sterile loofah mixture was cooled, *Lactiplantibacillus Plantarum* P101 (5%, m/V) was added and fermented at 37°C for 36 hours. After fermentation, it was centrifuged and the supernatant was aspirated to obtain the LF, which was placed in a −20°C refrigerator until use.

### Experimental animals and design

Skbex Biotechnology Co., Ltd. (Henan, China) provided us with 6 ~ 7 weeks old (18 ~ 20 g) of sexually mature female Balb/c mice. Mice were habituated to the laboratory animal room for one week before the experiment, during which they were supplied clean drinking water and appropriate food, a generally consistent temperature and humidity, and a 12 h light/12 h dark cycle.

**Fig. 1:**
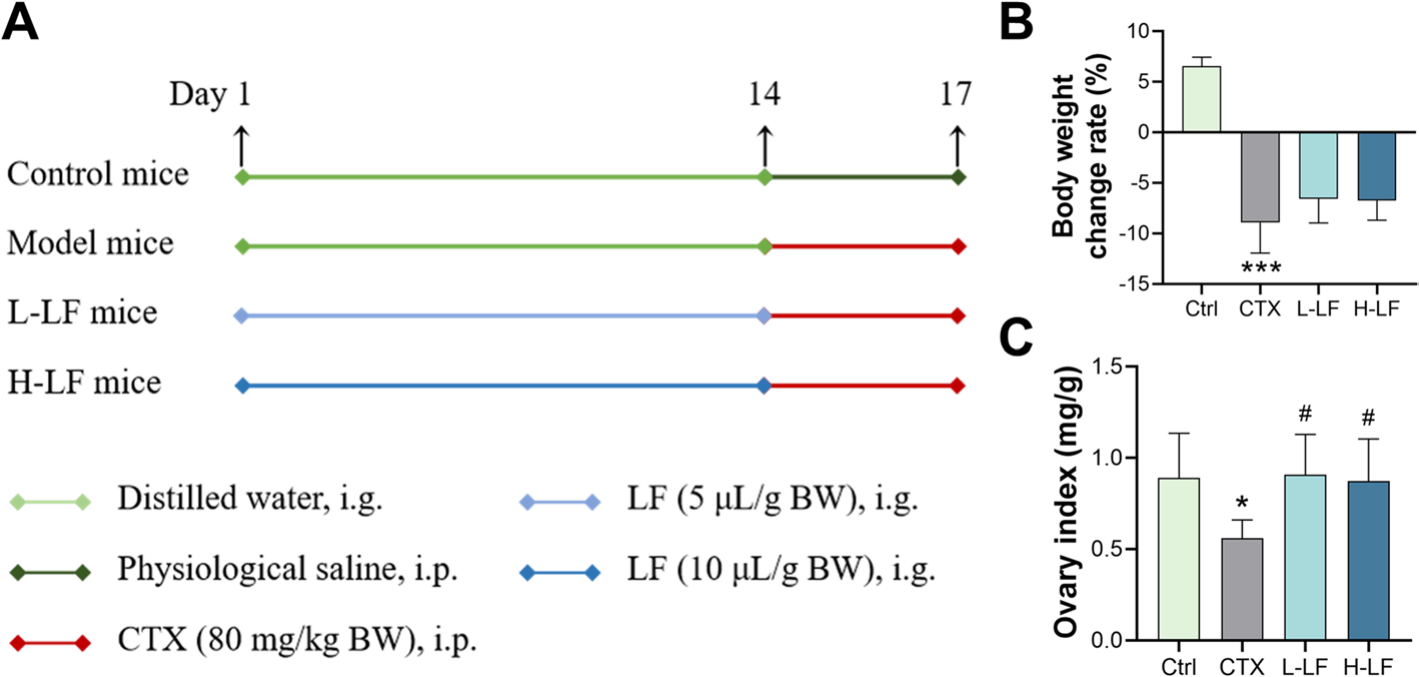
Effect of LF on body weight and ovarian index in mice exposed to CTX. **A** A schematic diagram showing the experimental design. **B** Body weight growth rate. **C** Ovary index of the four groups. *p < 0.05 and ***p < 0.001 (vs. control group); #*p* < 0.05 (vs. CTX group)

The mice were randomly divided into four groups (Fig. 1A, *n* = 8 per group): (1) control group, placebo; (2) model group, CTX, 80 mg/kg body weight (BW); (3) L-LF group, LF, 5 μL/g BW; and (4) H-LF group, LF, 10 μL/g BW. For 14 days, the mice in each treatment group were gavaged orally with the corresponding doses of LF, while the control and model groups were gavaged with an equal volume of distilled water. Mice in the control group were then injected intraperitoneally with physiological saline, whereas the mice in the other three groups were given CTX (80 mg/kg BW) via the same route of administration for three days, from day 15 to day 17. Their body weights were recorded every other day. Twenty-four hours after the last injection, all mice were sacrificed by neck dislocation, and the blood and ovaries were collected for further analysis. The ovary index was calculated as follows: ovary index (mg/g) = ovary weight/body weight.

### Histological morphology observation and follicle count

The ovaries were harvested from female mice and promptly immersed in the 4% paraformaldehyde solution. The samples were dehydrated using different ethanol gradients before being embedded in paraffin. Hematoxylin and eosin (H&E) were used to stain the paraffin sections with a thickness of 5 μm. The stained sections were then sealed with neutral gum. Finally, the sections were observed and photographed under the Nikon T*i* optical microscope (Tokyo, Japan).

Follicle stages were classified according to the previous definitions [20, 21] with minor modifications and counted: 1) primordial follicle: an oocyte surrounded by a single layer of flattened squamous follicular cells; 2) primary follicle: an oocyte surrounded by a single layer of cuboidal granulosa cells; 3) secondary follicle: 2 or more layers of cuboidal granulosa cells, without antrum; 4) antral follicle: follicle with an antral cavity, whatever the size. and 5) atretic follicle: with degenerating oocyte or pycnotic granulosa cells. The number of follicles in each stage was estimated by counting one section of each ovary. Data are presented as the number of follicles per developmental stage (*n* = 3 ovaries per group).

### ELISA assay and oxidative stress assay

The homogenate was prepared from ovary tissues. After centrifugation at 3000 rpm for 15 min, the supernatant of the tissue homogenate was obtained to assess the levels of interleukin-1β (IL-1β) and tumor necrosis factor-α (TNF-α) using ELISA kits (Shanghai YSRIBIO Industrial Co., Ltd., China). Assay kits were used to assess the protein content, activities of superoxide dismutase (SOD) and glutathione (GSH), as well as the level of malondialdehyde (MDA) in the ovaries (Nanjing Jiancheng, China). Whole blood samples were centrifuged to obtain the serum samples for the measurement of anti-Müllerian hormone (AMH), E2, FSH and luteinizing hormone (LH) by ELISA kits. The manufacturer’s instructions were followed for the operations of each parameter. A microplate reader (Thermo Scientific™ Varioskan™ LUX, USA) was used to determine absorbance values at specific wavelengths.

### Quantitative real-time polymerase chain reaction (RT-qPCR)

To begin, total RNA was extracted from the ovaries using the *Trelief*™ RNAprep FastPure Tissue&Cell Kit (TSINGKE Biotechnology Co., Ltd., China). Then, using the Hifair® II 1st Strand cDNA Synthesis Kit (YEASEN, Catalog# 11119ES60), the extracted RNA was reverse transcribed into cDNA. Following that, SYBR Green (TSINGKE, Catalog# TSE203) and the primer (Table 1, GENERAL Biosystems Co., Ltd., Anhui, China) were mixed with the template DNA strand. RT-qPCR was then operated on Agilent AriaMx Real-Time PCR System (Agilent Technologies, Inc., Singapore) under the following parameters: 1 cycle of 50°C for 2 min, 95°C for 2 min, 40 cycles of 95°C for 15 s, 55°C for 30 s and 72°C for 30 s. For each gene, the 2^-ΔΔCt^ method was used to determine the relative quantification of mRNA.

**Table 1:** Genes and primers used in the RT-qPCR

Gene	Primer	Sequence (5′ → 3′)
*TLR4*	Forward	CTGTATTCCCTCAGCACTCTTGATT
	Reverse	TGCTTCTGTTCCTTGACCCACT
*MyD88*	Forward	CGCATGGTGGTGGTTGTT
	Reverse	CGCTTCTGTTGGACACCT
*IKKα*	Forward	GTCAGGACCGTGTTCTCAAGG
	Reverse	GCTTCTTTGATGTTACTGAGGGC
*NF-κB*	Forward	ACACTGGAAGCACGGATGAC
	Reverse	TGTCTGTGAGTTGCCGGTCT
*TNF-α*	Forward	CTCATGCACCACCATCAAGG
	Reverse	ACCTGACCACTCTCCCTTTG
*IL-1β*	Forward	TTCAGGCAGGCAGTATCACTC
	Reverse	GAAGGTCCACGGGAAAGACAC
*IL-10*	Forward	CTTACTGACTGGCATGAGGATCA
	Reverse	GCAGCTCTAGGAGCATGTGG
*IL-4*	Forward	CCATATCCACGGATGCGACA
	Reverse	AAGCACCTTGGAAGCCCTAC
*Caspase-9*	Forward	AAGAAGACCGGAGTGCAATG
	Reverse	CATGACAGGATTATACAACCGC
*Caspase-3*	Forward	GGAGGCTGACTTCCTGTATGCTT
	Reverse	CCTGTTAACGCGAGTGAGAATG
*Bax*	Forward	GATGGCAACTTCAACTGGG
	Reverse	CCGAAGTAGGAGAGGAGGC
*Bcl-2*	Forward	CACTCGACCTTGTTTCTTCCAG
	Reverse	TCCTAACCCCTTGCTCTGCTT
*β-actin*	Forward	GCTCCTCCTGAGCGCAAGTA
	Reverse	CAGCTCAGTAACAGTCCGCC

### Immunohistochemistry (IHC) assay

The IHC analysis was used to determine the protein expression levels of toll-like receptor 4 (TLR4) and nuclear factor kappa B (NF-κB) p65 in the ovaries. First, paraffin sections of ovarian tissue were dewaxed by BioDewax and Clear Solution and then rehydrated with different concentrations of alcohol. Following that, sections were treated with antigen retrieval, endogenous peroxidase activity blocking, serum sealing, and rabbit polyclonal antibody incubation with TLR4 (1:1000, Catalog#GB 11519) and NF-κB p65 (1:400, Catalog#GB 13587). The sections were then incubated with a secondary antibody (Horseradish Peroxidase Conjugated Goat Anti-Rabbit IgG (H + L), Catalog#GB 23303), followed by the diaminobenzidine chromogenic reaction, nucleus counterstaining, dehydration, and mounting with SweSuper Clean BioMount Medium. Ultimately, all sections were photographed with an optical microscope. Using ImageJ 6.0, the IHC score was performed by measuring the average optical density in six high-power fields of at least three mice ovaries (National Institutes of Health).

### TUNEL assay

To detect ovarian cell apoptosis, we used terminal deoxyribonucleotide transferase-mediated nick end labeling (TUNEL) staining. First, ovarian paraffin sections were deparaffinized and rehydrated, antigen retrieved, permeabilized, and equilibrated at room temperature. Then, the sections were subjected to Tunel reaction, DAPI counterstaining in the nucleus, and coverslip with anti-fade mounting medium. Finally, the sections were observed under a fluorescent microscope. The assay was standardized with the use of an ovarian paraffin section treated with DNase I to induce the formation of DNA strand breaks (positive control of apoptosis). TUNEL-positive apoptotic cells (TUNEL+) had green nuclei, while TUNEL-negative apoptotic cells (TUNEL-) had blue nuclei. The number of apoptotic cells was determined by ImageJ 6.0. For each slide, five different fields of view were chosen at random, and apoptotic cells within the field of view were counted. Reagents required for IHC and TUNEL assays were obtained from Servicebio Technology Co., Ltd. (Hubei, China).

### Statistical analysis

The experimental data were processed to determine the mean and standard deviation (expressed as mean ± SD). In IBM SPSS Statistics 26, the one-way analysis of variance (ANOVA) and the least significant difference (LSD) test (SPSS, Inc., Chicago, Illinois) were used to compare the differences between groups. And statistical significance was determined to be *p* < 0.05.

## Results

### Effects of LF on body weight and ovarian index in the POF mice

The results showed that mice in the model, L-LF and H-LF groups had a significantly negative growth rate after CTX injection, whereas mice in the control group had a positive growth rate. Simultaneously, the model group’s ovarian index was significantly lower than the control group’s. The ovarian index prevented with L-LF and H-LF was significantly higher than in the model group (Fig. 1).

### LF improved the morphological alterations of ovary and sex hormone levels in the POF mice

**Fig. 2:**
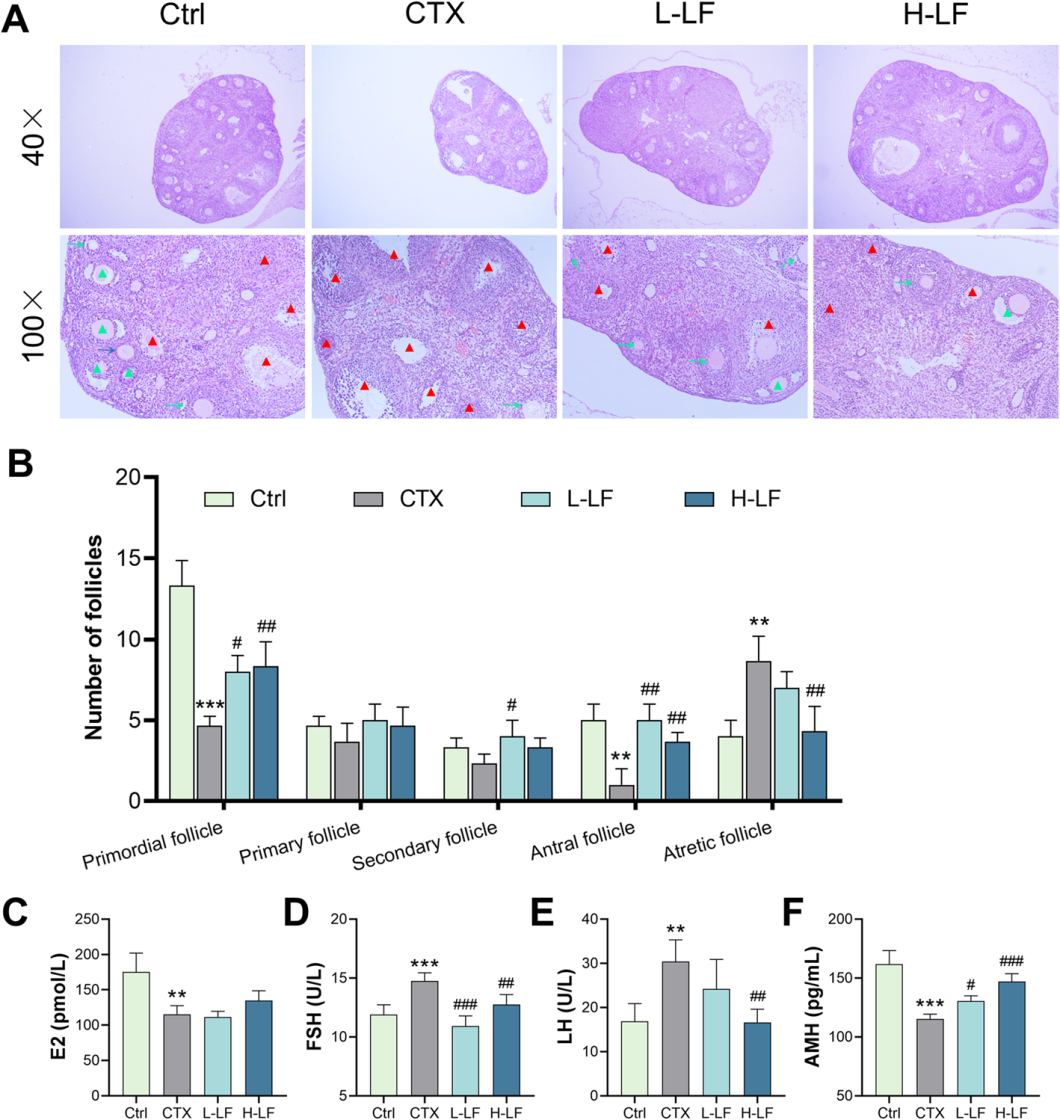
Effect of LF on ovarian histomorphology and serum sex hormone levels in mice exposed to CTX. **A** Sections of ovaries with H&E staining (× 40 and × 100 magnification). Blue arrows indicate primary follicles; green arrows indicate secondary follicles; green triangles indicate antral follicles; red triangles indicate atretic follicles. **B** The number of follicles in different stages. **C** The level of E2. **D** The level of FSH. **E** The level of LH. **F** The level of AMH. **p < 0.01 and ***p < 0.001 (vs. control group); #*p* < 0.05, ##*p* < 0.01 and ### *p* < 0.001 (vs. CTX group)

The histological morphology of mouse ovaries revealed that there were many follicles in various stages and a minimal number of atretic follicles in the control group. The ovaries of the model mice were much smaller, with a greater amount of atretic follicles and a limited amount of growing follicles, and the granulosa cells were disorganized. The granulosa cells of the follicles in the L-LF and H-LF groups were slightly disorganized, and there were many atretic follicles (Fig. 2A). The number of primordial and antral follicles in the ovaries of mice in the model group was significantly reduced, but the number of atretic follicles was significantly increased when compared to the control group, as seen in Fig. 2B. Pretreatment with both L-LF and H-LF doses significantly prevented the loss of primordial and antral follicles compared to the model group, and atretic follicle numbers were significantly reduced after H-LF treatment.

The serum levels of E2, FSH, LH, and AMH were measured by ELISA to assess the effect of LF on the sex hormones of POF mice (Fig. 2C-F). The results indicated that the levels of E2 and AMH were significantly lower and the levels of FSH and LH were significantly higher in the model group than the control group. However, both L-LF and H-LF pretreatment significantly decreased the level of FSH, and significantly increased the level of AMH compared with the model group. In addition, H-LF pretreatment also significantly reduced the level of LH (Fig. 2E).

### LF relieved ovary oxidative stress in the POF mice

**Fig. 3:**
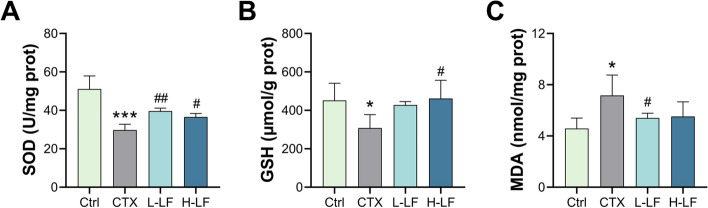
Effect of LF on oxidative stress state and the level of antioxidant enzyme in mice exposed to CTX. **A** The activity of SOD. **B** The content of GSH **C** The content of MDA. *p < 0.05 and ***p < 0.001 (vs. control group); #*p* < 0.05 and ##*p* < 0.01 (vs. CTX group)

We measured the levels of SOD, GSH and MDA in the ovaries to determine whether exposure of mice to CTX induces oxidative stress and the effects of LF on oxidative stress in the POF models (Fig. 3). The results indicated that the levels of SOD and GSH significantly decreased and the level of MDA significantly increased in the model group compared with the control group. However, when compared to the model group, L-LF pretreatment significantly increased the level of SOD and significantly decreased the level of MDA. Simultaneously, H-LF pretreatment significantly increased SOD and GSH levels.

### LF relieved the inflammation in the POF mice by inhibiting the TLR4/NF-κB signaling pathway

**Fig. 4:**
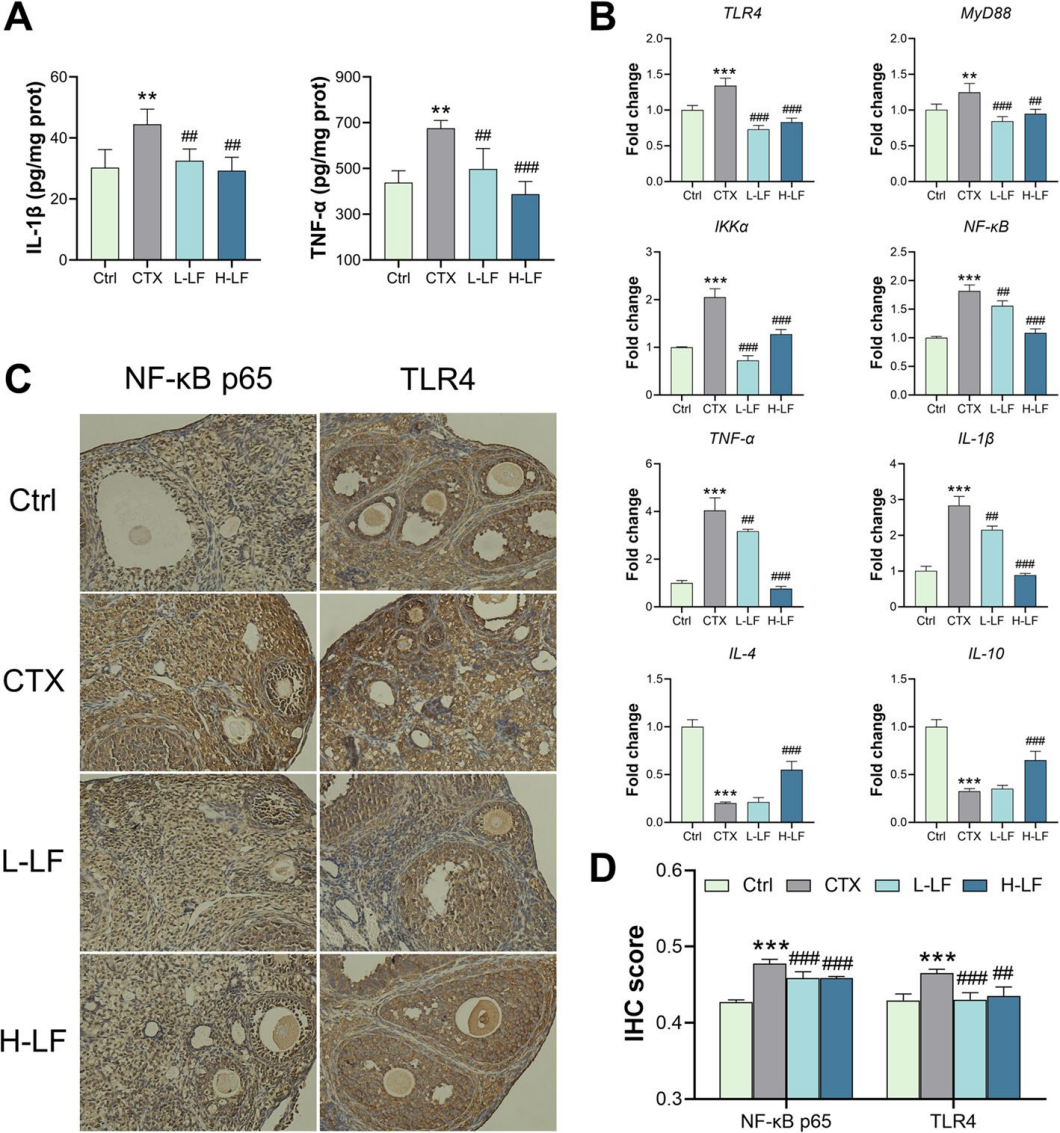
Effect of LF on inflammation in mice ovaries exposed to CTX. **A** The levels of inflammatory-related cytokines. **B** RT-qPCR results of TLR4/NF-κB pathway-related genes. **C** IHC images (× 200 magnification) of NF-κB p65 and TLR4. **D** IHC score of NF-κB p65 and TLR4. The nucleus of hematoxylin stained is blue, and the positive expression of DAB is brownish yellow. **p < 0.01 and ***p < 0.001 (vs. control group); ##*p* < 0.01 and ### *p* < 0.001 (vs. CTX group)

We evaluated the levels of TNF-α and IL-1β in the ovaries to determine whether exposure of mice to CTX induces inflammation and the effects of LF on inflammation in the POF models (Fig. 4A). The levels of TNF-α and IL-1β were found to be significantly higher in the model group than the control group. When compared with the model group, L-LF and H-LF pretreatment all significantly reduced the levels of TNF-α and IL-1β.

TLR4/NF-κB signaling pathway mRNA and protein expression levels were examined to explore the molecular mechanism of CTX-induced ovarian inflammation and dysfunction (Fig. 4). The TLR4/NF-κB signaling pathway-related genes *TLR4*, myeloiddifferentiationfactor88 (*MyD88*), IkappaB kinase-alpha (*IKKα*)*, NF-κB, TNF-α* and *IL-1β* were significantly upregulated, and anti-inflammatory genes interleukin-4 (*IL-4*) and interleukin-10 (*IL-10*) were significantly downregulated in the model group compared with the control group. *TLR4, MyD88, IKKα, NF-κB, TNF-α* and *IL-1β* genes were significantly down-regulated in L-LF and H-LF pretreated CTX-exposed mice, and *IL-4* and *IL-10* genes were significantly up-regulated in H-LF group mice compared with the model group. However, there was no significant difference in the transcription levels of the *IL-4* and *IL-10* genes between the L-LF group and the model group (Fig. 4B). Furthermore, IHC results indicated that the positive expressions of TLR4 and NF-κB p65 were significantly higher in the model group than the control group, whereas those in L-LF and H-LF pretreatment groups were significantly lower than the model group (Fig. 4C and D). These suggest that LF may alleviate CTX-induced ovarian inflammation by inhibiting the TLR4/NF-κB pathway.

### LF attenuated the apoptosis in the POF mice

**Fig. 5:**
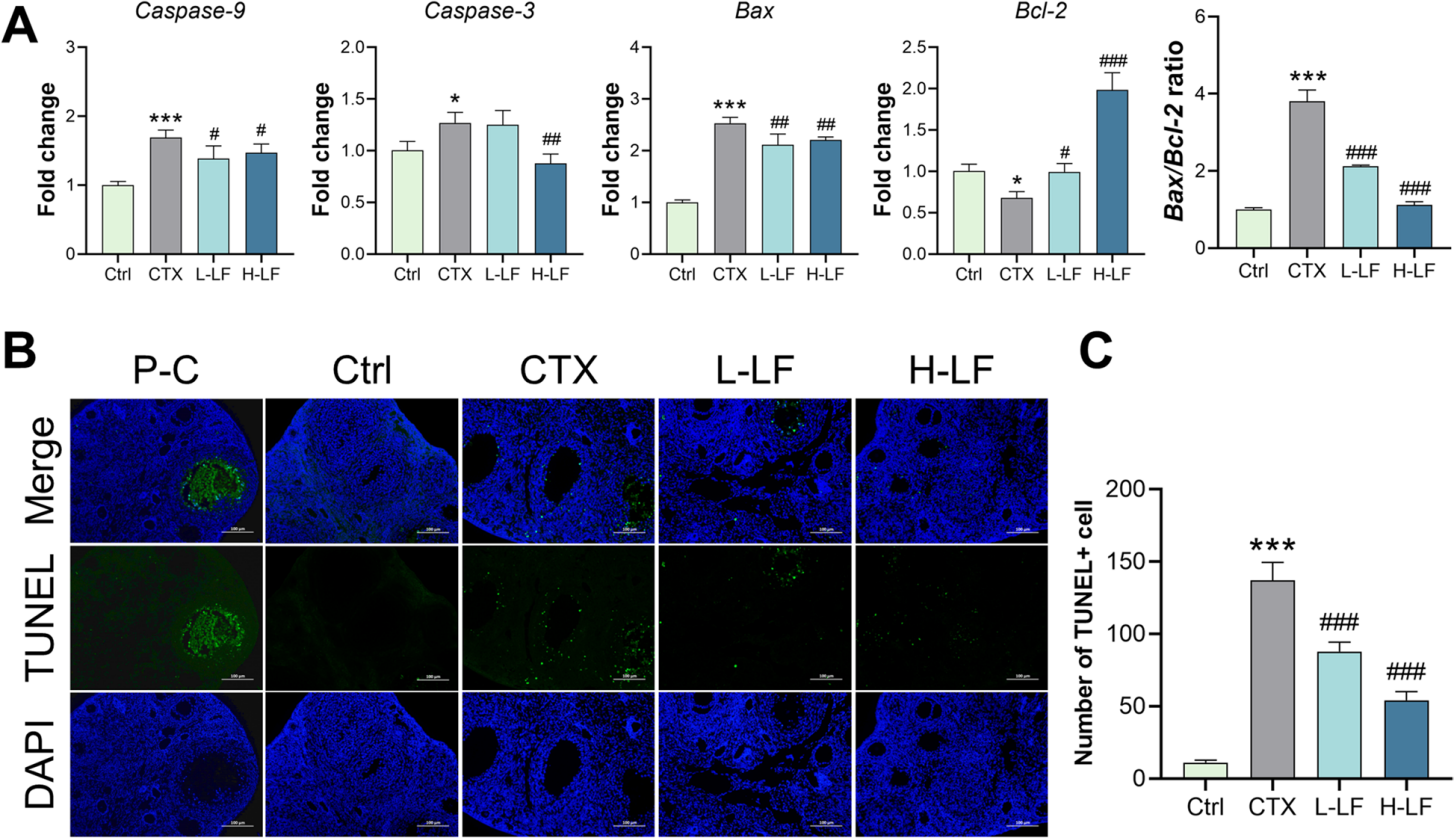
Effect of LF on apoptosis in mice ovaries exposed to CTX. **A** The mRNA levels of *Caspase-9*, *Caspase-3*, *Bax, Bcl-2* genes and the ratio of *Bax/Bcl-2* were detected by RT-qPCR. **B** Images of TUNEL and DAPI. The green stain indicates TUNEL+ apoptotic cells (× 200 magnification). P-C represents the positive control. **C** Quantitative analysis of TUNEL+ apoptotic cells. *p < 0.05 and ***p < 0.001 (vs. control group); #*p* < 0.05, ##*p* < 0.01 and ### *p* < 0.001 (vs. CTX group)

By measuring the mRNA levels of the ovarian apoptotic genes *Caspase-9, Caspase-3,* Bcl-2 associated X protein (*Bax*) and B-cell lymphoma-2 (*Bcl-2*), the mechanism through which LF regulated CTX-induced apoptosis was explored. The results showed that *Caspase-9, Caspase-3, Bax* and the ratio of *Bax/Bcl-2* genes were significantly up-regulated and the *Bcl-2* gene was significantly down-regulated in the model group compared with the control group. However, when compared with the model group, L-LF and H-LF pretreatment significantly down-regulated apoptotic genes *Caspase-9*, *Bax* and the ratio of *Bax/Bcl-2*, as well as up-regulated the *Bcl-2* gene*.* In addition, H-LF pretreatment also significantly down-regulated the apoptotic gene *Caspase-3* (Fig. 5A).

TUNEL staining results showed that there were relatively few TUNEL+ cells in the control group, while apoptotic cells were abundant in the model group. In addition, TUNEL+ cells were fewer in the L-LF and H-LF pretreatment groups than in the model group (Fig. 5B). Meanwhile, the quantitative analysis showed that the model group had a larger number of TUNEL+ cells than the control group. The number of TUNEL+ cells was significantly lower in L-LF and H-LF pretreated groups than in the model group. Furthermore, the H-LF group had fewer TUNEL+ cells than the L-LF group (Fig. 5C).

## Discussion

In young women, chemotherapy regimens increase the risk of POF and infertility. Therefore, we conducted experiments in vivo with the purpose of limiting the damaging effects of chemotherapeutic agents on the ovaries. In this study, we used CTX to induce the POF mouse model. CTX was found to cause significant atrophy of the ovaries in mice, with a significant reduction in the number of follicles at all levels and a growing number of atretic follicles, indicating that the POF model was successfully established.

In the current study, we discovered that LF administration enhanced the ovarian index, as well as the numbers of ovarian primordial follicles and antral follicles while decreasing the number of atretic follicles as compared to the model group. This data suggested that LF effectively enhanced folliculogenesis in POF mice. The development of POF is strongly connected to hormone levels. AMH belongs to the transforming growth factor b-super family and is generated by ovarian granulosa cells. It is a valid biological marker of ovarian reserve [22]. The reduction of AMH appears to be the earliest sign of ovarian damage following chemotherapy [23]. FSH and LH are crucial for the growth of follicles as well as the generation of E2 [24]. Monitoring E2, AMH, FSH, and LH in serum is therefore appropriate for detecting POF. Previous studies found that CTX lowered serum AMH and E2 levels while increasing FSH and LH levels [25, 26]. Treatment with LF significantly reversed these effects in our study, indicating that LF might increase ovarian reserve by preserving ovarian granulosa cells. Taken together, these data revealed the protective effect of LF against damage to follicle development caused by CTX. Besides, H-LF may have a better protective effect than L-LF.

Increasing data suggest that oxidative stress is important in the formation of POF [27]. To investigate the protective effects of LF on ovarian oxidative stress, we measured SOD, GSH and MDA, which are frequently used to evaluate the level of oxidative or anti-oxidative stress. In this research, the levels of SOD and GSH in the model group decreased significantly, while the level of MDA was significantly increased, indicating CTX induced strong oxidative stress as well as decreased antioxidant capacity of ovaries, which was similar to previous research [28]. However, LF reduced oxidative stress, indicating that LF could prevent CTX-induced ovarian oxidative stress.

We further investigated the underlying mechanisms relevant to the protective benefits of LF on mice with POF. Numerous molecular mechanisms are involved in the pathogenesis of cytotoxic chemotherapeutic agent-induced ovary toxicity, and one of the key mechanisms is the inflammatory response [29]. Inflammation plays a key physiological role in folliculogenesis and ovulation. However, abnormal inflammation could result in POF, decreased oocyte quality, and other related issues [30]. IL-1β and TNF-α are pro-inflammatory factors and are important cytokines for studying the inflammatory response. It has been demonstrated that CTX induced secretion of pro-inflammatory factors IL-1β and TNF-α [31]. In this work, we discovered that CTX stimulated an increase in IL-1β and TNF-α in the ovaries, while LF pretreatment alleviated the inflammatory response, and H-LF was more effective than L-LF. These suggested that LF exerts a preventive protective effect against CTX-induced inflammatory responses by inhibiting the secretion of pro-inflammatory factors, and H-LF may have better anti-inflammatory effects than L-LF.

The TLR4/NF-κB signaling pathway is the classical inflammatory signaling pathway [11]. The TLR4 activation triggers NF-κB activation, which leads to the transcription of pro-inflammatory cytokines and chemokines. This leads to ovulatory disruption by providing a pro-inflammatory environment in the ovary [32, 33]. Our present data found that LF reduced the expressions of critical genes and proteins in the TLR4/NF-κB signaling pathway in the ovaries of POF mice. These findings were similar to those from the previous literature [34–36]. It may be concluded that LF could prevent CTX-induced ovarian inflammation response via a TLR4/NF-κB dependent mechanism.

Interestingly, a study concluded that the presence of inflammation and oxidative stress could disrupt mitochondrial structural integrity, resulting in apoptosis [37]. Besides, studies have shown that follicular cyclic growth and development are mainly mediated by granulosa cells. Excessive apoptosis of granulosa cells could lead to accelerated follicular depletion in the form of follicular atresia, causing POF [38, 39]. The TUNEL analysis showed that the model group had more TUNEL+ cells than the control group in the current investigation. This was similar to previous findings [40, 41], indicating that CTX caused apoptosis in ovarian granulosa cells. Bcl-2 protein can block the release of apoptogenic proteins from mitochondria, hence preventing ovarian granulosa cell apoptosis [42]. Bax, on the other hand, is a pro-apoptotic protein that is important for the permeabilization of the outer mitochondrial membrane and the subsequent release of apoptogenic molecules, which results in caspase activation [43, 44]. We discovered that LF administration reduced mitochondrion-mediated apoptosis by down-regulating pro-apoptotic genes *Caspase-9, Caspase-3 and Bax*, and up-regulating the anti-apoptotic gene *Bcl-2*. It has been demonstrated that the Bax/Bcl-2 ratio may be more critical to determining apoptosis than either protein alone [45]. In this study, LF administration reduced the expression of the *Bax/Bcl-2* ratio, particularly in the H-LF group. The findings suggested that LF prevented apoptosis and increased cell survival in the ovaries. H-LF may be more effective in inhibiting apoptosis than L-LF.

Collectively, our study indicated that LF could ameliorate CTX-induced POF through anti-inflammatory, anti-oxidative stress, and anti-apoptosis. In addition, high doses of LF may be more effective than low doses of LF in CTX-induced POF mice.

## Conclusion

In summary, this study provided evidence that LF could significantly ameliorate hormonal imbalance, histopathological changes in ovaries, oxidative stress, and inflammation as well as apoptosis in female mice induced by CTX. It can be seen that LF might be a new approach that could prevent CTX-induced ovarian toxicity. This study has important implications for the development of probiotic fermented products for ovarian protection in terms of edible value. Moreover, LF pretreatment to CTX-induced ovarian injury exhibited a significant ameliorative effect probably via suppression of the TLR4/NF-κB and apoptotic pathways. More detailed research is needed to investigate the precise mechanisms.

## Data Availability

The data sets used and/or analyzed during the current study are available from the corresponding author upon reasonable request.

## Abbreviations

POF: Premature ovarian failure
CTX: Cyclophosphamide
LF: *Luffa cylindrica* fermentation liquid
FSH: Follicle stimulating hormone
LH: Luteinizing hormone
E2: Estradiol
AMH: Anti-Müllerian hormone
H&E: Hematoxylin and eosin
IL-1β: Interleukin-1β
TNF-α: Tumor necrosis factor-α
GSH: Glutathione
MDA: Malondialdehyde
RT-qPCR: Quantitative real-time polymerase chain reaction
IHC: Immunohistochemistry
TLR4: Toll-like receptor 4
NF-κB: Nuclear factor kappa B
TUNEL: Terminal deoxyribonucleotide transferase-mediated nick end labeling
MyD88: Myeloiddifferentiationfactor88
IKKα: IkappaB kinase-alpha
IL-4: Interleukin-4
IL-10: Interleukin-10
Bcl-2: B-cell lymphoma-2
Bax: Bcl-2 associated X protein
